# Epidemiology and association with outcomes of polypharmacy in patients undergoing surgery: retrospective, population-based cohort study

**DOI:** 10.1093/bjsopen/zrad041

**Published:** 2023-05-17

**Authors:** Freyja Jónsdóttir, Anna B Blöndal, Aðalsteinn Guðmundsson, Ian Bates, Jennifer M Stevenson, Martin I Sigurðsson

**Affiliations:** Pharmaceutical Sciences, University of Iceland, Reykjavik, Iceland; Pharmacy Services, Landspitali - The National University Hospital of Iceland, Reykjavik, Iceland; Pharmaceutical Sciences, University of Iceland, Reykjavik, Iceland; Development Centre for Primary Healthcare in Iceland, Primary Health Care of the Capital Area, Reykjavik, Iceland; Division of Geriatrics, Landspitali - The National University Hospital of Iceland, Reykjavik, Iceland; Faculty of Medicine, University of Iceland, Reykjavik, Iceland; School of Pharmacy, University College London, London, UK; Institute of Pharmaceutical Science, King's College, London, UK; Pharmacy Department, Guy’s and St Thomas’ NHS Foundation Trust, London, UK; Faculty of Medicine, University of Iceland, Reykjavik, Iceland; Division of Anaesthesia and Intensive Care Medicine, Landspitali - The National University Hospital of Iceland, Reykjavik, Iceland

## Abstract

**Background:**

The aim of this study was to determine the prevalence of preoperative polypharmacy and the incidence of postoperative polypharmacy/hyper-polypharmacy in surgical patients and their association with adverse outcomes.

**Methods:**

This was a retrospective, population-based cohort study among patients older than or equal to 18 years undergoing surgery at a university hospital between 2005 and 2018. Patients were categorized based on the number of medications: non-polypharmacy (fewer than 5); polypharmacy (5–9); and hyper-polypharmacy (greater than or equal to 10). The 30-day mortality, prolonged hospitalization (greater than or equal to 10 days), and incidence of readmission were compared between medication-use categories.

**Results:**

Among 55 997 patients, the prevalence of preoperative polypharmacy was 32.3 per cent (95 per cent c.i. 33.5 to 34.3) and the prevalence of hyper-polypharmacy was 25.5 per cent (95 per cent c.i. 25.2 to 25.9). Thirty-day mortality was higher for patients exposed to preoperative hyper-polypharmacy (2.3 per cent) and preoperative polypharmacy (0.8 per cent) compared with those exposed to non-polypharmacy (0.6 per cent) (*P* < 0.001). The hazards ratio (HR) of long-term mortality was higher for patients exposed to hyper-polypharmacy (HR 1.32 (95 per cent c.i. 1.25 to 1.40)) and polypharmacy (HR 1.07 (95 per cent c.i. 1.01 to 1.14)) after adjustment for patient and procedural variables. The incidence of longer hospitalization (greater than or equal to 10 days) was higher for hyper-polypharmacy (11.3 per cent) and polypharmacy (6.3 per cent) compared with non-polypharmacy (4.1 per cent) (*P* < 0.001). The 30-day incidence of readmission was higher for patients exposed to hyper-polypharmacy (10.2 per cent) compared with polypharmacy (6.1 per cent) and non-polypharmacy (4.8 per cent) (*P* < 0.001). Among patients not exposed to polypharmacy, the incidence of new postoperative polypharmacy/hyper-polypharmacy was 33.4 per cent (95 per cent c.i. 32.8 to 34.1), and, for patients exposed to preoperative polypharmacy, the incidence of postoperative hyper-polypharmacy was 16.3 per cent (95 per cent c.i. 16.0 to 16.7).

**Conclusion:**

Preoperative polypharmacy and new postoperative polypharmacy/hyper-polypharmacy are common and associated with adverse outcomes. This highlights the need for increased emphasis on optimizing medication usage throughout the perioperative interval.

**Registration number:**

NCT04805151 (http://clinicaltrials.gov).

## Teaser

Preoperative polypharmacy (5–9 medications) or hyper-polypharmacy (greater than or equal to 10 medications) was common (58 per cent) and had an association with adverse outcomes, highlighting the importance of medication optimization.

## Introduction

Annually, over 300 million surgical procedures are performed worldwide^1^, and this number is expected to grow in the coming decades^2,3^. The surgical population is ageing at a higher rate than the general population, resulting in a significant growth in demand for surgical services. To optimize clinical outcomes for surgical patients, it is essential to identify subgroups at increased risk of poorer outcomes^2^.

One such subgroup consists of surgical patients exposed to polypharmacy, the simultaneous use of multiple medications^4,5^. The most widely accepted definition for polypharmacy is the use of five or more medications^6–8^, and, recently, hyper-polypharmacy, ten or more medications, has been introduced^9^. Polypharmacy and hyper-polypharmacy are associated with: increased risk of frailty^7^; reduced medication adherence^10^; increased likelihood of unplanned hospitalization^7,10^; loss of functional ability^10^; increased risk of drug interactions^10^; increased usage of healthcare resources^10^; and greater mortality^7^. One of the goals of the WHO is an increased focus on patients exposed to polypharmacy, in order to optimize their medications and reduce harm due to medication usage^11^. Another potential focus group is patients using multidose drug dispensing services, where medications are dispensed into one unit for each administration. The use of multidose drug dispensing services has increased, and there is growing evidence regarding its relation to polypharmacy^12^ and suboptimal medication appropriateness^13–15^.

While polypharmacy may be rational in individual patients with multiple diseases, its prevalence has been used as a quality indicator of prescribing practices^16^. Polypharmacy has been identified as the leading risk for inappropriate prescribing practices^16–18^. This stresses the importance of assessing medication appropriateness among patients exposed to polypharmacy to ensure that treatment is safe and effective.

Many studies on polypharmacy focus on the older population, specific medication classes^19^, or general practice^20^. There is a lack of knowledge regarding the incidence of polypharmacy in the adult surgical population, although a recent study demonstrated that polypharmacy is associated with functional decline in older cardiac surgery patients^4^.

The aim of this study was to determine the prevalence of preoperative polypharmacy and the incidence of postoperative polypharmacy/hyper-polypharmacy and their association with patient and procedural variables. Furthermore, the authors studied the association between preoperative polypharmacy and postoperative outcomes.

It was hypothesized that preoperative and postoperative polypharmacy is common, especially among older patients, patients with a high co-morbidity and frailty burden, and patients undergoing more complicated surgery. It was further hypothesized that preoperative polypharmacy and hyper-polypharmacy is associated with increased short- and long-term mortality, a longer primary hospitalization, and a higher risk of readmission.

## Methods

### Study population

This study was a retrospective, population-based cohort study that included all patients older than or equal to 18 years undergoing their first surgery at Landspitali - The National University Hospital of Iceland, during the study interval, between December 2005 and December 2018. The hospital performs all tertiary surgeries and serves as the primary hospital for all surgery for most of the nation.

Ethical approval was obtained from the National Bioethics Committee of Iceland (VSN-14-139-V1) and the Data Protection Authority of Iceland. All databases used for research were de-identified before statistical analysis, and all work was compliant with the General Data Protection Regulation of the European Union. The study protocol was published on clinicaltrials.gov before analysis (NCT04805151)^21^, and the study reporting adheres to the STROBE guidelines for reporting of observational studies in epidemiology^20^.

### Clinical data

This study used the Icelandic perioperative database, a retrospective database that includes clinical data on all surgical procedures performed at Landspitali. Database assembly has been described previously^19^. The database contains information on surgery type and anatomical location using the Nordic Medico-Statistical Committee (NOMESCO) Classification of Surgical Procedures (NCSP; version 1.14) for surgical classifications^22^. Patient co-morbidities were registered based on ICD9/10 coding from primary care and hospital. The co-morbidity burden was described by calculating the Charlson co-morbidity index and the Elixhauser co-morbidity index, and the frailty risk was assessed by using the hospital frailty risk score^23^. Adverse reactions were defined as any documentation of ICD9/10 codes for adverse drug effect (Y40–59, X40–59, T36–59). Information on filled medications was from the Prescription Medicines Registry of the Directorate of Health database. This national electronic database includes real-time information about all outpatient drug prescriptions in Iceland. Its accuracy is determined regularly by comparing prescribed medications against dispensed medications and is estimated to be 95 per cent. The database includes all prescribed regular and as-required medications, but does not include over-the-counter, topical, and herbal medications. This study obtained data on filled prescriptions 1 year before surgery and up to 1 year after surgery and whether a multidose drug dispensing service was used^19^.

### Exposure variable definition

The primary exposure was the extent of medication use, defined as the number of different medications filled in the year preceding surgery (preoperative) and the year after surgery (postoperative). Patients were also grouped into categories of non-polypharmacy (fewer than 5), polypharmacy (5–9), and hyper-polypharmacy (greater than or equal to 10) based on their preoperative and postoperative polypharmacy. Furthermore, for each individual, the numbers of medications within different anatomical/pharmacological groups (Anatomical Therapeutic Chemical (ATC) first level) and pharmacological/therapeutical subgroups (ATC second level) were counted in the year preceding surgery and the year after surgery.

### Outcome data

The following outcomes were considered: short- and long-term mortality; long primary hospital stay (greater than or equal to 10 days); and risk of readmission (fewer than 30 days).

### Statistical analysis

Data analysis was conducted from May 2021 to October 2021. Statistical analyses were performed using R (The R Foundation for Statistical Computing, Vienna, Austria) version 4.0.3, via R studio (RStudio PBC, Boston, MA, USA), version 1.4.1106. Descriptive statistics were used to present the number of medications. The distribution of the group into preoperative and postoperative medication-use categories was described as a percentage with a 95 per cent c.i. calculated using Pearson–Klopper in the binom package in R. Logistic regression was used to compare univariate and multivariate patient and procedural variables between groups of varying preoperative and postoperative medication use.

Adverse outcomes were compared between categories of medication use using chi-squared tests. Similarly, adverse outcomes were compared between patients with and without an increase in polypharmacy from the year preceding surgery to the year after surgery (increase from no polypharmacy to polypharmacy/hyper-polypharmacy or polypharmacy to hyper-polypharmacy). The association between long-term survival and risk of readmission was plotted using Kaplan–Meier methods and modelled using a Cox proportional hazard risk model. The proportionality assumption was assessed using the cox.zph function in R, quantifying changes in Schoenfeld residuals against time.

To visualize the relationship between 30-day mortality, 30-day readmission, and long primary hospital stay (greater than or equal to 10 days), and the number of medications filled in the year preceding surgery, a restricted cubic spline analysis was performed, with predefined knots of 0, 5, and 10 to mimic the polypharmacy and hyper-polypharmacy classes. No missing data were identified in the variables used for this study.

## Results

### Clinical characteristics of the patient cohort by preoperative filling

**Fig. 1:**
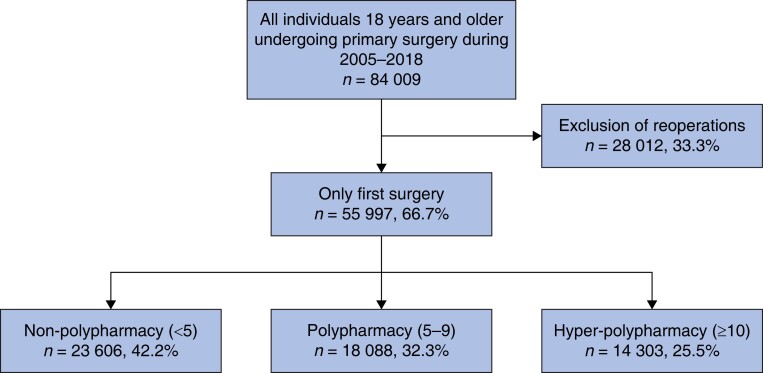
CONSORT diagram of participant inclusion based on the number of medications filled in the year preceding surgery (fewer than 5 medications = non-polypharmacy, 5–9 medications = polypharmacy, and greater than or equal to 10 medications = hyper-polypharmacy)

The cohort included 55 997 surgical patients 18 years and older. Of the cohort, 32 136 were female (57.0 per cent), and the median age was 55 (interquartile range (i.q.r.) 39–69) years. Of those, 23 606 (42.2 per cent (95 per cent c.i. 41.7 to 42.6)), 18 988 (32.3 per cent (95 per cent c.i. 33.5 to 34.3)), and 14 303 (25.5 per cent (95 per cent c.i. 25.2 to 25.9)) experienced preoperative non-polypharmacy (fewer than 5), polypharmacy (5–9), and hyper-polypharmacy (greater than or equal to 10) respectively (*Fig. 1*). *Table 1* compares the patient characteristics, including co-morbidities and medication usage, of the patient cohort based on varying degrees of polypharmacy. Most surgeries were elective (65.8 per cent), and elective surgeries were more common among patients exposed to polypharmacy and hyper-polypharmacy than non-polypharmacy. For the cohort, orthopaedic surgical procedures were most common, followed by abdominal, gynaecological, and neurological procedures. Hypertension was the most common co-morbidity among patients exposed to hyper-polypharmacy (55.8 per cent) and polypharmacy (35.0 per cent). However, a benign neoplasm was most common among patients exposed to non-polypharmacy (*Table 1*). With increasing preoperative polypharmacy, there was a higher median age and a higher proportion of female patients. There was also a higher underlying burden of co-morbidity and frailty risk measured by composite indices and individual diagnoses.

**Table 1:** Patient characteristics of the patient cohort based on the number of different medications filled in the year preceding surgery (fewer than 5 medications = non-polypharmacy, 5–9 medications = polypharmacy, and greater than or equal to 10 medications = hyper-polypharmacy)

	Non-polypharmacy	Polypharmacy	Hyper-polypharmacy	All patients	*P*
Total patients	23 606 (42.2)	18 088 (32.3)	14 303 (25.5)	55 997	
Female	12 310 (52.1)	10 806 (59.7)	9020 (63.1)	32 136 (57.4)	<0.001
Age (years), median (i.q.r.)	45.00 (32.00−59.00)	58.00 (43.00−69.00)	67.00 (55.00−76.00)	55.00 (39.00−69.00)	<0.001
Multidose dispensing services before surgery	916 (3.9)	2148 (11.9)	4616 (32.3)	7680 (13.7)	<0.001
Number of preoperative medications, median (i.q.r.)	2.00 (1.00−3.00)	7.00 (6.00−8.00)	13.00 (11.00−16.00)	6.00 (2.00−10.00)	<0.001
Number of postoperative medications, median (i.q.r.)	3.00 (1.00−5.00)	7.00 (5.00−10.00)	13.00 (9.00−17.00)	6.00 (3.00−11.00)	<0.001
Elixhauser co-morbidity index*, median (i.q.r.)	0.00 (0.00−3.00)	0.00 (0.00−4.00)	3.00 (0.00−8.00)	0.00 (0.00−4.00)	<0.001
**Hospital frailty risk score class**					
Low (<5)	18 096 (76.7)	10 586 (58.5)	5034 (35.2)	33 716 (60.2)	
Medium (5–15)	5201 (22.0)	6894 (38.1)	7402 (51.8)	19 497 (34.8)	
High (>15)	309 (1.3)	608 (3.4)	1867 (13.1)	2784 (5.0)	
**Co-morbidities**					<0.001
Ischaemic heart disease	952 (4.0)	2416 (13.4)	4248 (29.7)	7616 (13.6)	
Congestive heart failure	220 (0.9)	425 (2.3)	1358 (9.5)	2003 (3.6)	
Hypertension	2787 (11.8)	6330 (35.0)	7976 (55.8)	17 093 (30.5)	
Diabetes mellitus	334 (1.4)	1026 (5.7)	2360 (16.5)	4381 (7.8)	
Chronic obstructive pulmonary disease	1814 (7.7)	2839 (15.7)	4323 (30.2)	8976 (16.0)	
Liver disease	147 (0.6)	223 (1.2)	361 (2.5)	731 (1.3)	
Chronic kidney disease	128 (0.5)	316 (1.7)	961 (6.7)	1405 (2.5)	
Malignant neoplasm	2632 (11.1)	3093 (17.1)	3343 (23.4)	9068 (16.2)	
Benign neoplasm	4444 (18.8)	5007 (27.7)	5657 (39.6)	15 108 (27.0)	
Psychiatric	1759 (7.5)	2139 (11.8)	3003 (21.0)	6901 (12.3)	
Delirium	449 (1.9)	674 (3.7)	1020 (7.1)	2143 (3.8)	
**Surgery location and classification**					<0.001
Emergency operation	10 247 (43.4)	5072 (28.0)	3841 (26.9)	19 160 (34.2)	
Abdominal	4781 (20.3)	3415 (18.9)	2439 (17.1)	10 635 (18.9)	
Cardiac	499 (2.1)	726 (4.0)	596 (4.2)	1821 (3.3)	
Endocrine	464 (2.0)	340 (1.9)	238 (1.7)	1042 (1.9)	
Gynaecology	4450 (18.8)	2978 (16.5)	1469 (10.3)	8897 (15.9)	
Neurosurgery	2309 (9.8)	2335 (12.9)	1770 (12.4)	6414 (11.4)	
Orthopaedic	6983 (29.6)	4490 (24.9)	4221 (29.5)	15 694 (28.1)	
Thoracic	417 (1.8)	306 (1.6)	386 (2.7)	1109 (2.0)	
Urology	1397 (5.9)	1468 (8.1)	1307 (9.1)	4172 (7.4)	
Vascular	1335 (5.6)	1243 (6.9)	1142 (8.0)	3720 (6.7)	

Values are *n* (%) unless otherwise indicated. *The Elixhauser co-morbidity index is a severity index to quantify various patient co-morbidities from multiple chronic diseases into a single number that can be used to assess and correct for patient co-morbidity burden. i.q.r., interquartile range.

The authors assessed reclassification of polypharmacy classification if a shorter window of time to fill before surgery was considered (*Fig. S1*). This revealed that, for example, if only the last 6 months before surgery were considered to classify polypharmacy, roughly 60 per cent of the patients would remain within their medication-use category compared with a 12-month filling window. Similarly, if antibiotics were removed from the list of medications, 80.2 per cent of patients exposed to polypharmacy and 79.9 per cent exposed to hyper-polypharmacy would have remained within their medication-use category.

### Types of medications used and multidose dispensing

The most common classes of medications filled before surgery for the whole group were antibiotics (49.0 per cent), cardiac medications (42.4 per cent), and opioids (42.2 per cent) (*Table S1*). For the group with preoperative hyper-polypharmacy, the most commonly filled medication classes were cardiac medications (77.8 per cent), followed by antibiotics (75.0 per cent) and opioids (67.0 per cent). Similarly, patients exposed to polypharmacy most commonly filled antibiotics (56.4 per cent), followed by cardiac medications (50.9 per cent) and opioids (46.8 per cent), and patients exposed to non-polypharmacy most commonly filled antibiotics (27.7 per cent), followed by opioids (23.6 per cent) and paracetamol/orphenadrine combinations (20.7 per cent) in the year preceding surgery.

Of the 55 997 patients, 7680 (13.7 per cent) used a multidose drug dispensing service before surgery. Patients who used multidose drug dispensing services before surgery were older, more likely to undergo cardiac and orthopaedic surgery, and had a higher burden of major co-morbidities and frailty risk, including diagnoses affecting cognitive function (delirium, dementia, and psychiatric diagnoses). They were also more likely to have a previous diagnosis of an adverse drug reaction (*Table S2*). After surgery, 8.1, 22.7, and 69.1 per cent of patients exposed to non-polypharmacy, polypharmacy, and hyper-polypharmacy utilized multidose drug dispensing services respectively.

### Incidence of new postoperative polypharmacy/hyper-polypharmacy

Of 23 606 patients who were not exposed to preoperative polypharmacy, the incidence of new postoperative polypharmacy/hyper-polypharmacy was 33.4 per cent (95 per cent c.i. 32.4 to 34.0), and the incidence of new postoperative hyper-polypharmacy was 16.3 per cent (95 per cent c.i. 16.0 to 16.7). For patients exposed to polypharmacy, the incidence of new postoperative hyper-polypharmacy was 28.9 per cent (95 per cent c.i. 28.3 to 29.6). Patients who had an increase in their number of medications, moving from either non-polypharmacy to polypharmacy or polypharmacy to hyper-polypharmacy were older and had a longer hospital stay compared with those with no change. They had a lower Elixhauser co-morbidity index and a lower hospital frailty risk index classification and were more likely to have a malignant neoplasm (23.3 *versus* 14.0 per cent) (*P* < 0.001). Additionally, these patients were more likely to have undergone cardiac surgery (6.4 *versus* 2.3 per cent) (*P* < 0.001) or vascular surgery (10.9 *versus* 5.5 per cent) (*P* < 0.001) (*Table S3*).

### Clinical outcomes of patients with varying preoperative medication use

**Fig. 2:**
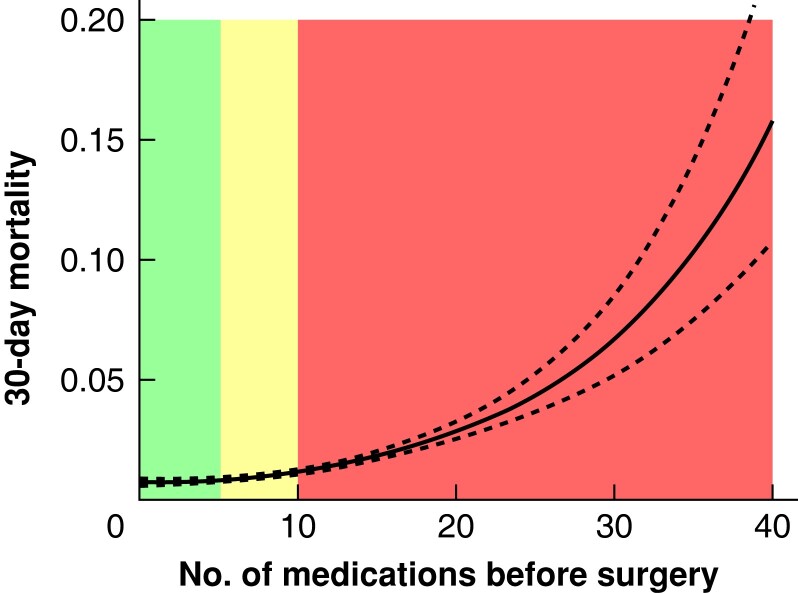
Association between the number of medications before surgery and 30-day mortality The figure shows the result of restricted cubic spline analysis of the proportion of patients with the three outcomes. Colours indicate the number of different medications filled in the year preceding surgery: green, fewer than 5 medications = non-polypharmacy; yellow, 5–9 medications = polypharmacy; and red, greater than or equal to 10 medications = hyper-polypharmacy.

**Fig. 3:**
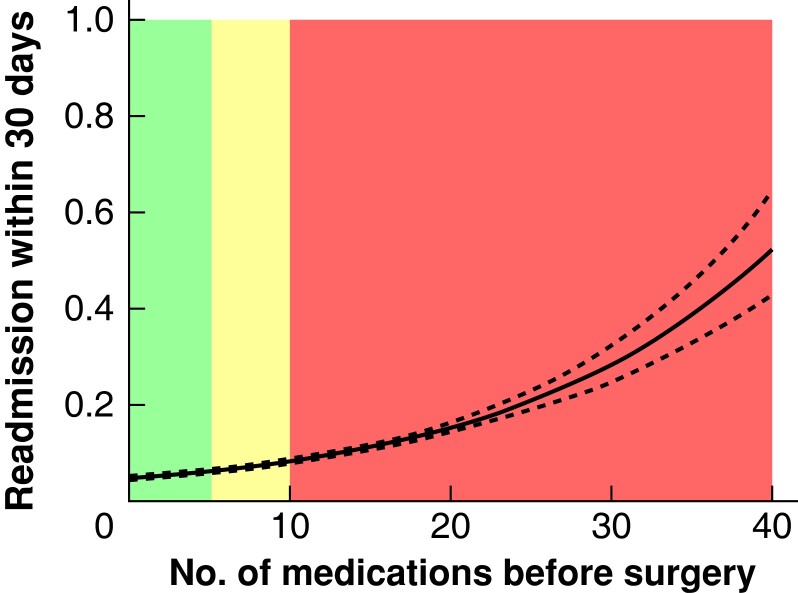
Association between the number of medications before surgery and the risk of readmission within 30 days The figure shows the result of restricted cubic spline analysis of the proportion of patients with the three outcomes. Colours indicate the number of different medications filled in the year preceding surgery: green, fewer than 5 medications = non-polypharmacy; yellow, 5–9 medications = polypharmacy; and red, greater than or equal to 10 medications = hyper-polypharmacy.

**Fig. 4:**
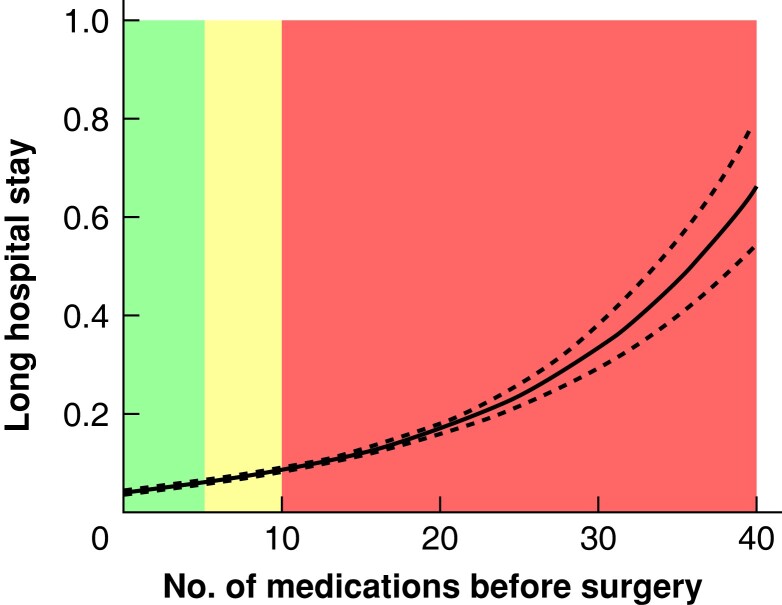
Association between the number of medications before surgery and a long primary hospital stay (greater than or equal to 10 days) The figure shows the result of restricted cubic spline analysis of the proportion of patients with the three outcomes. Colours indicate the number of different medications filled in the year preceding surgery: green, fewer than 5 medications = non-polypharmacy; yellow, 5–9 medications = polypharmacy; and red, greater than or equal to 10 medications = hyper-polypharmacy.

An unadjusted restricted cubic spline analysis revealed a strong relationship between the absolute number of different medications filled in the year preceding surgery and the incidence of 30-day mortality, the risk of readmission within 30 days, and a long primary hospital stay (*Fig. 2*, *Fig. 3*, and *Fig. 4*). Thirty-day mortality for patients exposed to preoperative hyper-polypharmacy was 2.3 per cent, compared with 0.8 and 0.6 per cent for patients exposed to polypharmacy and non-polypharmacy respectively (*P* < 0.001). A long primary hospital stay (greater than or equal to 10 days) was more common for patients exposed to hyper-polypharmacy (11.3 per cent) and polypharmacy (6.3 per cent) compared with non-polypharmacy (4.1 per cent) (*P* < 0.001). Similarly, the incidence of 30-day readmission was higher for patients exposed to hyper-polypharmacy (10.2 per cent) compared with polypharmacy (6.1 per cent) and non-polypharmacy (4.8 per cent) (*P* < 0.001). Patients who were readmitted within 30 days had a higher incidence of a diagnosis of adverse drug reactions within 30 days compared with those who did not get readmitted (1 *versus* 0.1 per cent) (*P* < 0.001).

### Long-term survival

**Fig. 5:**
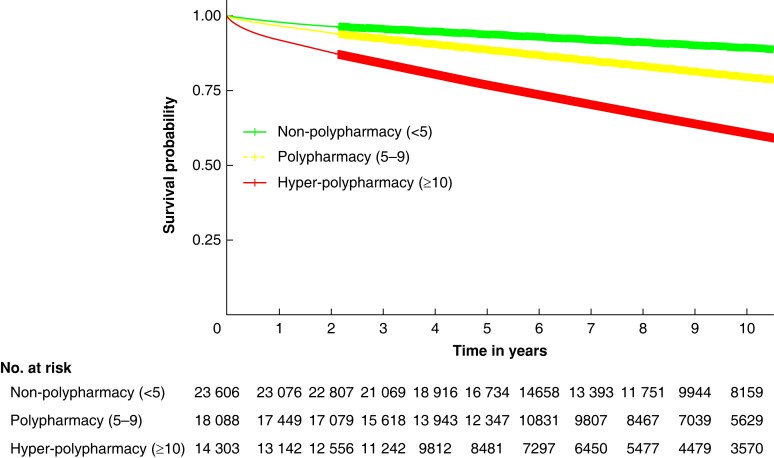
Survival of cohort based on polypharmacy classification A Kaplan–Meier survival curve of long-term survival of patients compared based on the number of medications before surgery (green, fewer than 5 medications = non-polypharmacy; yellow, 5–9 medications = polypharmacy; and red, greater than or equal to 10 medications = hyper-polypharmacy). Thicker lines represent 95% confidence intervals.

*Fig. 5* compares long-term survival between groups of variable polypharmacy classification with 1-year all-cause mortality. After adjustment for age, sex, length of stay, co-morbidities (hypertension, diabetes, chronic obstructive pulmonary disease, ischaemic heart disease, liver disease, chronic kidney disease, malignant neoplasm, and benign neoplasm), Elixhauser co-morbidity index, procedural classification and urgency, there was a higher hazards ratio (HR) of long-term mortality for patients exposed to hyper-polypharmacy (HR 1.32 (95 per cent c.i. 1.25 to 1.40)) and polypharmacy (HR 1.07 (95 per cent c.i. 1.01 to 1.14)) compared with non-polypharmacy (HR 1.00 (reference)).

## Discussion

This study identified that preoperative polypharmacy/hyper-polypharmacy and new postoperative polypharmacy/hyper-polypharmacy were common among surgical patients, especially older patients, with a high co-morbidity and frailty risk burden. In addition, the findings confirm that preoperative polypharmacy is associated with a higher short- and long-term mortality, a longer primary hospital stay, and a higher risk of readmission.

Previous studies have investigated polypharmacy, and the prevalence and incidence vary among countries, although there appears to be an overall rise in the trend of polypharmacy^7,24–29^. These studies are often difficult to compare due to the lack of a coherent definition of polypharmacy and variation in the study population.

Most literature regarding polypharmacy uses similar definitions, but has a focus on the epidemiology in a general population and mostly in older patients^4,5,25–27,29^. In a Scottish study of patients from a general population (older than or equal to 20 years), the prevalence of polypharmacy was 16.3 per cent and that of hyper-polypharmacy was 5.8 per cent^30^. Similarly, a study of general practice patients (older than or equal to 18 years) in Switzerland found that the prevalence of either polypharmacy or hyper-polypharmacy was 24 per cent^28^. Likewise, a Danish study of general practice older adults (older than or equal to 65 years) found the prevalence of polypharmacy and hyper-polypharmacy to be 29.0 and 5 per cent respectively^26^. Finally, a Swedish study of general practice older adults (older than or equal to 65 years) found the prevalence of polypharmacy to be 44.0 per cent and that of hyper-polypharmacy to be 11.7 per cent^27^. All of these are substantially lower than our reported rates of polypharmacy (32.3 per cent) and hyper-polypharmacy (25.5 per cent). However, it should be kept in mind that the authors describe a surgical population that likely has a higher disease burden, in particular in the year preceding surgery. Indeed, the observed prevalence of preoperative polypharmacy and hyper-polypharmacy is close to the reported prevalence in two studies conducted in surgical populations, although the target populations were older. A Canadian study on elective non-cardiac surgery patients (older than 65 years) reported a 54.8 per cent prevalence of polypharmacy^5^ and a study from the Netherlands on older patients (older than 70 years) undergoing cardiac surgery reported the prevalence of polypharmacy and hyper-polypharmacy to be 67 and 26 per cent^4^.

The authors found that the incidence of new postoperative polypharmacy in the year after surgery was 33.4 per cent, and new hyper-polypharmacy was 6.7 per cent among patients not exposed to preoperative polypharmacy, and the incidence of new postoperative hyper-polypharmacy for patients exposed to preoperative polypharmacy was 28.9 per cent. A Danish study of older adults (older than or equal to 65 years) estimated the 5-year incidence of polypharmacy to be 46.9 per cent and that of hyper-polypharmacy to be 17.7 per cent, slightly lower than identified in the current study. It should be kept in mind that the population and follow-up time were different^26^. These findings raise questions about whether surgery or admission to hospital may be a gateway into new or an accelerated rate of polypharmacy or hyper-polypharmacy.

Patient factors associated with polypharmacy and hyper-polypharmacy were not unexpected, as older patients are more likely to have multiple co-morbidities potentially requiring medications^7,30^. Previous studies have shown similar results^31^. Interestingly, a high hospital frailty risk score class is associated with polypharmacy, but not hyper-polypharmacy, potentially due to the patients being more sensitive to adverse drug effects and a shift towards deprescribing in this patient cohort.

It was found in the current study that the rate of multidose drug dispensing service utilization increased with levels of polypharmacy, co-morbidity burden, and age. Multidose drug dispensing services may be convenient for patients taking multiple medicines, especially those with worse cognitive function. Annual renewal of prescriptions for a multidose drug dispensing service may also give a unique platform periodically for medication optimization if used appropriately. Unfortunately, the use of multidose drug dispensing services also makes it more challenging to deprescribe, and increased automation in renewal could potentially discourage deprescribing. Studies of older adults (older than 75 years) have identified multidose drug dispensing services as a risk factor for uncritical renewals and insufficient medication optimization^13,27^. This subgroup of patients therefore warrants special attention to ensure their medications are optimally managed, both before surgery and after surgery.

The most common classes of prescriptions filled in the year preceding surgery for the whole group were antibiotics (49.0 per cent), cardiac medications (42.4 per cent), and opioids (42.2 per cent). The high prescription rates for antibiotics and opioids are concerning. The high usage rate of antibiotics raises questions about overuse and should be researched further due to increasing concern regarding the development of antibiotic resistance. Even though a high prevalence of opioid users was expected in a surgical population that is predominantly awaiting elective surgery, it is likely that these patients might benefit from medication counselling during their wait for surgery to reduce the risk for persistent postoperative opioid use and the harmful effects from prolonged opioid usage^19,32^.

The clinical outcomes associated with degree of polypharmacy were largely consistent with the previous literature documenting the association of polypharmacy with adverse clinical outcomes^5,7,25,26,29^. Despite a clear dose–response relationship and a biological plausibility, it is unclear whether these adverse clinical outcomes are directly mediated by polypharmacy, such as by adverse drug effects, or if they serve as a marker of co-morbidity burden only. While causality cannot be established, a potential mechanism explaining the connection between polypharmacy and the risk of readmission and mortality could be mediated through a risk of inappropriate prescribing that sets a patient at risk of increased anticholinergic burden, at risk of falls via orthostatism, and at risk of respiratory complications. This is a topic of further research.

One of the study’s strengths is that it makes use of a centralized nationwide Prescription Medicines Registry that allows detailed information to be obtained; it includes over 95 per cent of all prescriptions in the country, and the ability to link different registries to collect information via the personal identification number. A key strength is the large number of participants included in an extensive surgical database and complete follow-up for survival analysis. Another strength is that all surgeries were performed at the same national hospital.

A notable limitation is the dependence of classifying polypharmacy burden based on the number of different ACT classes filled. Using this method may overestimate the number of medications the participants take regularly. It might include medications solely used in the perioperative interval like antibiotics and opioids, which might inflate the number of medications and increase the incidence. However, it also does not include over-the-counter medications, which can contribute to polypharmacy. Also, combination therapies (such as combinations of angiotensin-converting-enzyme inhibitors and diuretics), count as a single medication in the data set. For particular subgroups of patients, such as patients with hypertension, the methodology underestimates the degree of polypharmacy^33^. It is not possible to state that the readmission, mortality, or longer hospital stay was directly related to medication-related problems.

While the presence of polypharmacy/hyper-polypharmacy certainly associates with a higher burden of co-morbidity and frailty, this is highly associated with the potential for inappropriate prescribing and could be used to identify patients who would benefit from medication review. A special emphasis should be put on medications listed as potentially inappropriate for older patients based on criteria such as the Beers criteria or screening tool for older persons prescriptions/screening tool to alert to right treatment criteria and the appropriate use of antibiotics, pain medications, and sedatives in the perioperative interval. Additionally, patients at risk of new postoperative polypharmacy could be identified and guided towards targeted follow-up focused on medication review and deprescribing, ideally within a multidisciplinary team involving clinical pharmacists, primary care physicians, or geriatricians when appropriate.

## Supplementary Material

zrad041_Supplementary_Data

## Data Availability

The study permissions do not allow individual patient data sharing.
