# Supplementary material for: Epidemiology and association with outcomes of polypharmacy in patients undergoing surgery: retrospective, population-based cohort study
Source: BJS Open. 2023 May 17;7(3):zrad041. doi: 10.1093/bjsopen/zrad041 (PMC10189279; doi:10.1093/bjsopen/zrad041)
Supplement: zrad041_Supplementary_Data [file zrad041_supplementary_data.docx]

**Epidemiology and association with outcomes of polypharmacy in patients undergoing surgery: retrospective, population-based cohort study**

Freyja Jónsdóttir^1,2^, Anna Bryndís Blöndal^1,3^, Aðalsteinn Guðmundsson^4,5^, Ian Bates^6^, Jennifer M Stevenson^7,8^, Martin I. Sigurðsson^5,9^

^1^ Pharmaceutical Sciences, University of Iceland, Reykjavik, Iceland

^2^ Pharmacy Services, Landspitali – The National University Hospital of Iceland, Reykjavik, Iceland

^3^ Development Centre for Primary Healthcare in Iceland, Reykjavik, Iceland

^4^ Division of Geriatrics, Landspitali – The National University Hospital of Iceland, Reykjavik, Iceland

^5^ Faculty of Medicine, University of Iceland, Reykjavik, Iceland

^6^ School of Pharmacy, University College London, United Kingdom

^7^ Institute of Pharmaceutical Science, King’s College, London, United Kingdom

^8^ Pharmacy Department, Guys and St Thomas’ NHS Foundation Trust, London, United Kingdom

^9^ Division of Anaesthesia and Intensive Care Medicine, Landspitali -The National University Hospital of Iceland, Reykjavik, Iceland

Corresponding Author: Freyja Jónsdóttir, Pharmaceutical Sciences, University of Iceland, Reykjavik, Iceland. Email: [freyjaj@hi.is](mailto:freyjaj@hi.is). ORCID ID: <https://orcid.org/0000-0002-9232-6723>

**Supplementary Materials – Index**

| **Supplementary Figures and Tables** |  |
| --- | --- |
| Figure S1 | *pag. 2* |
| Table S1 | *pag. 3* |
| Table S2  Table S3 | *pag. 4-5*  *pag.6* |
|  |  |


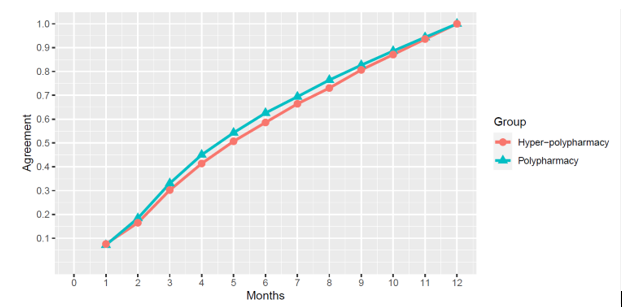


### Figure S1

Proportion of classification agreement (Y-axis) for patients classified into polypharmacy or hyper-polypharmacy groups when the study definition of including medications filled in the 12 months preceding surgery was compared against reclassification using a shorter duration of filling (1-11 months (X-axis)).

### Table S1

The table shows the patients' patterns of preoperatively prescribed medications. The number of medications pre-surgery stratifies patient (<5 medications = non-polypharmacy, 5-9 medications = polypharmacy and ≥ 10 medications = hyper-polypharmacy). Values are presented as n (%) or median (IQR) unless specified otherwise.

|  | Non-Polypharmacy | Polypharmacy | Hyper_Polypharmacy | All patients | p |
| --- | --- | --- | --- | --- | --- |
| Total number of patients | 23606 | 18088 | 14303 | 55997 |  |
| Pre-operative medication |  |  |  |  | <0.001 |
| Proton Pump Inhibitors | 1720 (7.3) | 4390 (24.3) | 7368 (51.5) | 13478 (24.1) |  |
| Anti-diabetics | 198 ( 0.8) | 975 ( 5.4) | 2216 ( 15.5) | 3389 ( 6.1) |  |
| Anticoagulants | 377 (1.6) | 1966 (10.9) | 4004 (28.0) | 6347 (11.3) |  |
| Antiplatelet | 204 (0.9) | 1231 (6.8) | 2685 (18.8) | 4120 (7.4) |  |
| Cardiac | 3417 (14.5) | 9198 (50.9) | 11126 (77.8) | 23741 (42.4) |  |
| Beta-blockers | 1211 (5.1) | 4378 (24.2) | 6386 (44.6) | 11975 (21.4) |  |
| Calcium Channel Blockers | 395 (1.7) | 2006 (11.1) | 3552 (24.8) | 5953 (10.6) |  |
| ACE inhibitors and Angiotensin II Receptor Blockers | 1566 (6.6) | 4737 (26.2) | 6305 (44.1) | 13156 (23.4) |  |
| Statins | 885 (3.7) | 3595 (19.9) | 5677 (39.7) | 10157 (18.1) |  |
| Urinary | 4007 (17.0) | 6449 (35.7) | 6606 (46.2) | 17062 (30.5) |  |
| Hormones | 1247 (5.3) | 3783 (20.9) | 6566 (45.9) | 11596 (20.7) |  |
| Corticosteroids | 640 (2.7) | 2444 (13.5) | 5024 (35.1) | 8108 (14.5) |  |
| Antibiotics | 6530 (27.7) | 10202 (56.4) | 10724 (75.0) | 27456 (49.0) |  |
| Opioids | 5568 (23.6) | 8469 (46.8) | 9582 (67.0) | 23619 (42.2) |  |
| Paracetamol/orphenadrine combinations | 4877 (20.7) | 7930 (43.8) | 7570 (52.9) | 20377 (36.4) |  |
| Nonsteroidal anti-inflammatory drugs | 4609 (19.5) | 7263 (40.2) | 6547 (45.8) | 18419 (32.9) |  |
| Selective cox-2 inhibitors | 316 (1.3) | 1037 (5.7) | 1821 (12.7) | 3174 (5.7) |  |
| Antipsychotic | 259 (1.1) | 988 (5.5) | 2052 (14.3) | 3299 (5.9) |  |
| Benzodiazepines | 723 (3.1) | 2586 (14.3) | 4952 (34.6) | 8261 (14.8) |  |
| Antidepressants | 1617 (6.8) | 4112 (22.7) | 6317 (44.2) | 12046 (21.5) |  |
| Anti-dementia | 40 (0.2) | 162 (0.9) | 284 (2.0) | 486 (0.9) |  |
| Respiratory | 2180 (9.2) | 5317 (29.4) | 7401 (51.7) | 14898 (26.6) |  |
| Antihistamin | 591 (2.5) | 1529 (8.5) | 2522 (17.6) | 4642 (8.3) |  |

### Table S2

Patient characteristics of cohorts (<5 medications = non-polypharmacy, 5-9 medications = polypharmacy and ≥ 10 medications = hyper-polypharmacy) based on the number of medications filled in the year preceding surgery and whether they used multidose dispensing services. Values are presented as n (%) or median (IQR) unless specified otherwise

|  | Non-polypharmacy - no multidose dispensing | Non-polypharmacy - multidose dispensing | Polypharmacy - no multidose dispensing | Polypharmacy - multidose dispensing | Hyper polypharmacy - no multidose dispensing | Hyper polypharmacy - multidose dispensing | p |
| --- | --- | --- | --- | --- | --- | --- | --- |
| Total number of patients | 22690 | 916 | 15940 | 2148 | 9687 | 4616 |  |
| Sex (female) | 11875 (52.3) | 435 (47.5) | 9636 (60.5) | 1170 (54.5) | 6274 (64.8) | 2746 (59.5) | <0.001 |
| Age (%) |  |  |  |  |  |  | <0.001 |
| 15-24 | 2839 (12.5) | 27 (2.9) | 913 (5.7) | 36 (1.7) | 226 (2.3) | 29 (0.6) |  |
| 25-34 | 4299 (18.9) | 59 (6.4) | 1649 (10.3) | 67 (3.1) | 487 (5.0) | 100 (2.2) |  |
| 35-44 | 4445 (19.6) | 46 (5.0) | 2217 (13.9) | 79 (3.7) | 830 (8.6) | 145 (3.1) |  |
| 4-54 | 4179 (18.4) | 54 (5.9) | 2802 (17.6) | 115 (5.4) | 1397 (14.4) | 262 (5.7) |  |
| 55-64 | 3608 (15.9) | 116 (12.7) | 3531 (22.2) | 234 (10.9) | 2384 (24.6) | 544 (11.8) |  |
| 65-74 | 2127 (9.4) | 141 (15.4) | 3080 (19.3) | 427 (19.9) | 2595 (26.8) | 1060 (23.0) |  |
| 75-84 | 883 (3.9) | 275 (30.0) | 1483 (9.3) | 692 (32.2) | 1551 (16.0) | 1549 (33.6) |  |
| 85-94 | 281 (1.2) | 169 (18.4) | 258 (1.6) | 458 (21.3) | 210 (2.2) | 877 (19.0) |  |
| >95 | 29 (0.1) | 29 (3.2) | 7 (0.0) | 40 (1.9) | 7 (0.1) | 50 (1.1) |  |
| Adverse drug reaction preoperative (%) | 643 (2.8) | 55 (6.0) | 864 (5.4) | 195 (9.1) | 979 (10.1) | 681 (14.8) | <0.001 |
| Adverse drug reaction preoperative (%) | 510 (2.2) | 52 (5.7) | 733 (4.6) | 143 (6.7) | 742 (7.7) | 493 (10.7) | <0.001 |
| Number of preoperative medications (median [IQR]) | 1.98 (1.43) | 2.33 (1.48) | 6.73 (1.39) | 7.16 (1.39) | 13.80 (4.04) | 15.84 (5.24) | <0.001 |
| Number of postoperative medications (median [IQR]) | 3.74 (3.39) | 6.34 (4.74) | 7.38 (4.26) | 9.31 (4.61) | 12.50 (6.05) | 15.13 (6.88) | <0.001 |
| **Elixhauser Comorbidity Index** |  |  |  |  |  |  | <0.001 |
| (<1] | 16853 (74.3) | 433 (47.3) | 9567 (60.0) | 852 (39.7) | 4388 (45.3) | 1318 (28.6) |  |
| (1-4] | 3598 (15.9) | 174 (19.0) | 3273 (20.5) | 375 (17.5) | 2057 (21.2) | 716 (15.5) |  |
| (4-5] | 673 (3.0) | 59 (6.4) | 804 (5.0) | 165 (7.7) | 626 (6.5) | 318 (6.9) |  |
| (5-8] | 819 (3.6) | 118 (12.9) | 1071 (6.7) | 283 (13.2) | 928 (9.6) | 568 (12.3) |  |
| (>8] | 747 (3.3) | 132 (14.4) | 1225 (7.7) | 473 (22.0) | 1688 (17.4) | 1696 (36.7) |  |
| **Hospital Frailty Risk Score Class** |  |  |  |  |  |  | <0.001 |
| Low (< 5) | 17663 (77.8) | 433 (47.3) | 9818 (61.6) | 768 (35.8) | 4054 (41.8) | 980 (21.2) |  |
| Med (5-15) | 4811 (21.2) | 390 (42.6) | 5823 (36.5) | 1071 (49.9) | 5018 (51.8) | 2384 (51.6) |  |
| High (> 15) | 216 (1.0) | 93 (10.2) | 299 (1.9) | 309 (14.4) | 615 (6.3) | 1252 (27.1) |  |
| **Comorbidities** |  |  |  |  |  |  |  |
| Hypertension | 2496 (11.0) | 291 (31.8) | 5206 (32.7) | 1124 (52.3) | 4939 (51.0) | 3037 (65.8) | <0.001 |
| Diabetes Mellitus | 279 (1.2) | 55 (6.0) | 806 (5.1) | 220 (10.2) | 1293 (13.3) | 1067 (23.1) | <0.001 |
| Chronic obstructive pulmonary disease | 1703 (7.5) | 111 (12.1) | 2455 (15.4) | 384 (17.9) | 2738 (28.3) | 1585 (34.3) | <0.001 |
| Ischemic heart disease | 796 (3.5) | 156 (17.0) | 1870 (11.7) | 546 (25.4) | 2314 (23.9) | 1934 (41.9) | <0.001 |
| Liver disease | 133 (0.6) | 14 (1.5) | 187 (1.2) | 36 (1.7) | 195 (2.0) | 166 (3.6) | <0.001 |
| Chronic kidney disease | 100 (0.4) | 28 (3.1) | 195 (1.2) | 121 (5.6) | 394 (4.1) | 567 (12.3) | <0.001 |
| Malignant neoplasm | 2438 (10.7) | 194 (21.2) | 2651 (16.6) | 442 (20.6) | 2225 (23.0) | 1118 (24.2) | <0.001 |
| Benign neoplasm | 4211 (18.6) | 233 (25.4) | 4358 (27.3) | 649 (30.2) | 3622 (37.4) | 2035 (44.1) | <0.001 |
| Delerium | 345 (1.5) | 104 (11.4) | 396 (2.5) | 278 (12.9) | 367 (3.8) | 653 (14.1) | <0.001 |
| Dementia | 65 (0.3) | 61 (6.7) | 11 (0.1) | 129 (6.0) | 18 (0.2) | 197 (4.3) | <0.001 |
| Psychiatric | 1501 (15.5) | 1502 (32.5) | 1527 (6.7) | 232 (25.3) | 1573 (9.9) | 566 (26.4) | <0.001 |
| **Surgery Location and Classification** |  |  |  |  |  |  | <0.001 |
| Emergency operation | 9719 (42.8) | 528 (57.6) | 3982 (25.0) | 1090 (50.7) | 2021 (20.9) | 1820 (39.4) | <0.001 |
| Abdominal | 4685 (20.6) | 96 (10.5) | 3148 (19.7) | 267 (4.6) | 459 (12.5) | 651 (14.1) |  |
| Cardiac | 438 (1.9) | 61 (6.7) | 590 (3.7) | 136 (6.3) | 381 (3.9) | 215 (4.7) |  |
| Endocrine | 452 (2.0) | 12 (1.3) | 319 (2.0) | 21 (1.0) | 179 (1.8) | 59 (1.3) |  |
| Gynaecology | 4413 (19.4) | 37 (4.1) | 2889 (18.1) | 89 (4.2) | 1255 (12.9) | 214 (4.6) |  |
| Neurosurgery | 2227 (9.8) | 82 (8.9) | 2140 (13.4) | 195 (9.1) | 1323 (13.7) | 447 (9.7) |  |
| Orthopaedic | 6547 (28.8) | 436 (47.6) | 3488 (21.9) | 1002 (46.6) | 2362 (24.4) | 1859 (40.5) |  |
| Thoracic | 403 (1.7) | 14 (1.6) | 276 (1.8) | 31 (1.4) | 276 (2.9) | 110 (2.4) |  |
| Urology | 1326 (5.9) | 71 (7.7) | 1286 (8.1) | 182 (8.4) | 864 (9.0) | 443 (9.6) |  |
| Vascular | 1267 (5.6) | 68 (7.4) | 1102 (7.0) | 141 (6.6) | 761 (7.9) | 381 (8.2) |  |

### Table S3

Patient characteristics of cohorts based on whether they changed to a higher polypharmacy category when medication use in the year preceding was compared with the medication use in the year following discharge from surgery. Values are presented as n (%) or median (IQR) unless specified otherwise.

|  | No change in polypharmacy category | Shift to higher polypharmacy category | p |
| --- | --- | --- | --- |
| Total number of patients | 42872 | 13125 |  |
| Sex (female) | 24744 (57.7) | 7392 (56.3) | 0.005 |
| Age, (median [IQR]), years | 55.00 [38.00, 69.00] | 57.00 [42.00, 68.00] | <0.001 |
| Length of stay (days)(median [IQR]) | 1.00 [0.00, 3.00] | 2.00 [1.00, 5.00] | <0.001 |
| Number of preoperative medications (median [IQR]) | 6.00 [2.00, 11.00] | 4.00 [2.00, 7.00] | <0.001 |
| Number of postoperative medications (median [IQR]) | 5.00 [2.00, 9.00] | 10.00 [6.00, 12.00] | <0.001 |
| **Elixhauser Comorbidity Index [IQR])** | 2.18 (5.38) | 2.18 (4.83) | 0.936 |
| **Hospital Frailty Risk Score Class** |  |  | <0.001 |
| Low (< 5) | 25428 (59.3) | 8288 (63.1) |  |
| Med (5-15) | 14994 (35.0) | 4503 (34.3) |  |
| High (> 15) | 2450 (5.7) | 334 (2.5) |  |
| **Surgery Location and Classification** |  |  | <0.001 |
| Emergency operation | 15131 (35.3) | 4029 (30.7) | <0.001 |
| Abdominal | 8369 (19.5) | 2266 (17.3) |  |
| Cardiac | 976 (2.3) | 845 (6.4) |  |
| Endocrine surgery | 751 (1.8) | 291 (2.2) |  |
| Gynaecology | 7015 (16.4) | 1882 (14.4) |  |
| Neurosurgery | 5331 (12.4) | 1083 (8.3) |  |
| Orthopaedic | 12245 (28.6) | 3449 (26.2) |  |
| Thoracic | 757 (1.8) | 352 (2.7) |  |
| Urology | 3225 (7.5) | 947 (7.2) |  |
| Vascular | 2285 (5.5) | 1435 (10.9) |  |
| **Comorbidities** |  |  |  |
| Congestive heart failure | 1674 ( 3.9) | 329 ( 2.5) | <0.001 |
| Ischemic heart disease | 5891 (13.7) | 1725 (13.1) | 0.083 |
| Hypertension | 13124 (30.6) | 3969 (30.2) | 0.424 |
| Diabetes Mellitus | 3098 (7.2) | 622 (4.7) | <0.001 |
| Chronic obstructive pulmonary disease | 7177 (16.7) | 1799 (13.7) | <0.001 |
| Liver disease | 582 (1.4) | 149 (1.1) | 0.055 |
| Chronic kidney disease | 1190 (2.8) | 215 (1.6) | <0.001 |
| Malignant neoplasm | 6009 (14.0) | 3059 (23.3) | <0.001 |
| Benign neoplasm | 11728 (27.4) | 3380 (25.8) | <0.001 |
| Psychiatric | 5564 (13.0) | 1337 (10.2) | <0.001 |
| Delirium | 1750 (4.1) | 393 (3.0) | <0.001 |
| Adverse drug reaction | 2774 (6.5) | 643 (4.9) | <0.001 |

^c^ The Elixhauser comorbidity index is a severity index to quantify various patient comorbidities from multiple chronic diseases into a single number that can be used to assess and correct for patient comorbidity burden.

Values are presented as count (%) or median (IQR) unless specified otherwise.
